# Double Delayed Enhancement: Concomitant Cardiac Amyloidosis and Acute Coronary Embolism

**DOI:** 10.14797/mdcvj.294

**Published:** 2021-08-26

**Authors:** Talha Ahmed, Nils P. Johnson, Anju Bhardwaj, Rafael Francisco C. Go, Mina F. Hanna, Bihong Zhao, Danai Kitkungvan

**Affiliations:** 1 The University of Texas Health Science Center at Houston, Houston, Texas, US; 2 Memorial Hermann Heart & Vascular Institute, Houston, Texas, US

**Keywords:** cardiac amyloidosis, cardiac magnetic resonance imaging, coronary angiography, coronary embolism, delayed enhancement

## Abstract

Hereditary cardiac amyloidosis (CA) is a relatively rare cause of nonischemic cardiomyopathy. The risk of intracardiac thrombi increases significantly in patients with CA. We report a case of a patient presenting with chest pain and acute myocardial infarction who was subsequently diagnosed with concomitant CA and acute coronary embolism.

## Case Presentation

**Figure 1:**
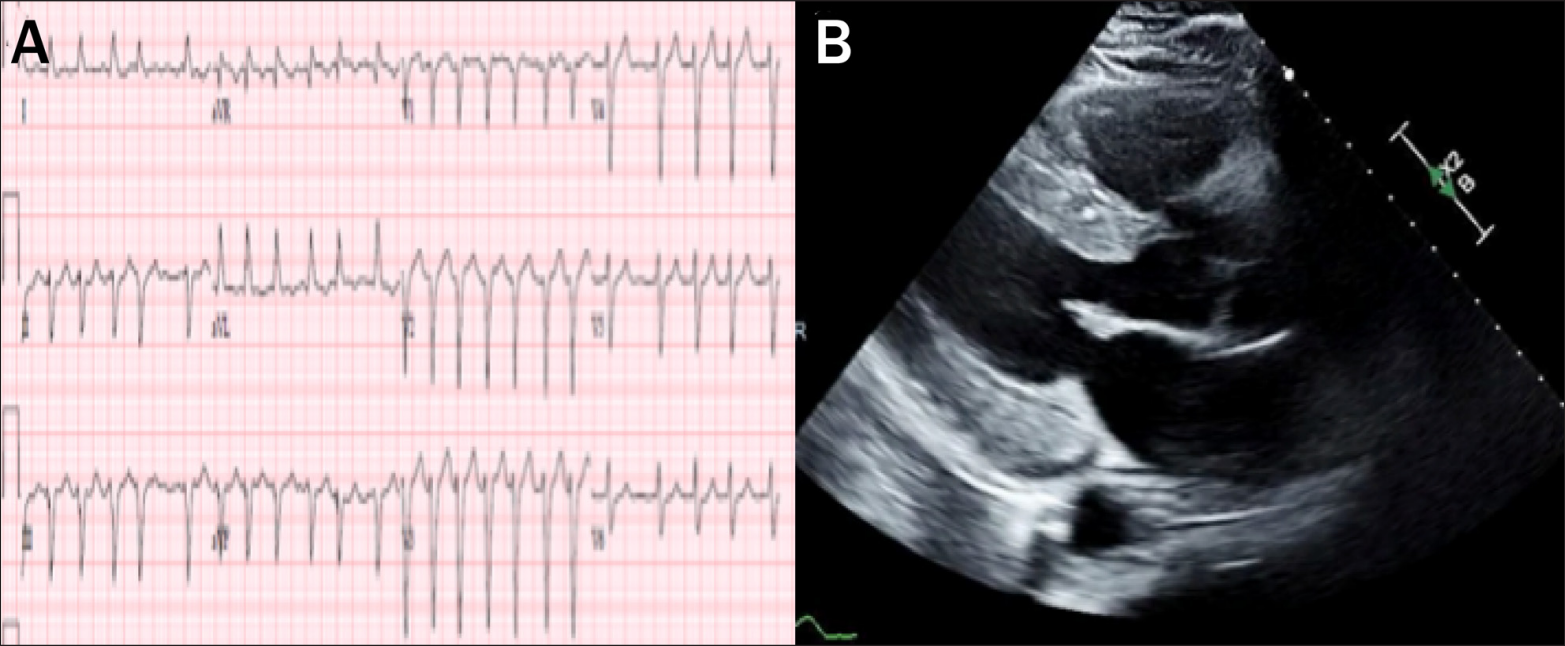
(**A**) Electrocardiography at presentation showed atrial fibrillation with rapid ventricular response but no evidence of low QRS voltage. (**B**) Echocardiography showed severe global hypokinesis of the left ventricle with a concentric wall thickness.

**Figure 2:**
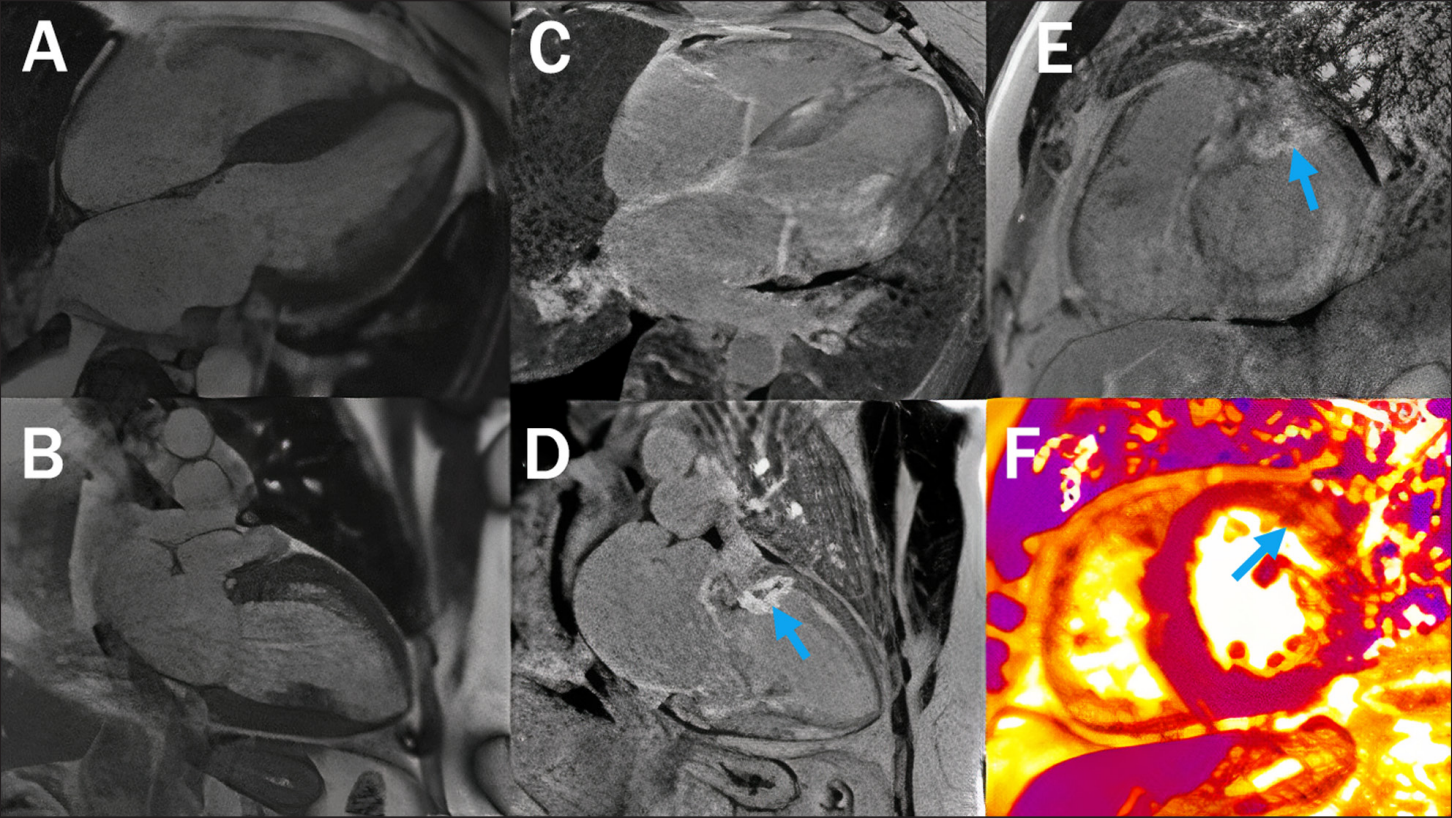
Cardiovascular magnetic resonance imaging showed (**A, B**) diffusely increased left ventricular wall thickness with (**C–E**) diffuse patchy late gadolinium enhancement (LGE) in the left ventricle and both atria consistent with cardiac amyloidosis. (**D, E,** arrow) There also is dense focal transmural LGE in the basal anterior and anterolateral wall with microvascular obstruction at its center and myocardial edema noted on T2 mapping images (**F**, arrow) that suggest acute myocardial infarction in a ramus or diagonal artery distribution. Image enlarged using letsenhance.io.

A 59-year-old Black male presented with acute chest pain, and examination revealed an irregular pulse. His medical history was notable for atrial fibrillation (AF), which was being treated with apixaban, and nonischemic cardiomyopathy with prior hypertension and diabetes mellitus. A coronary angiography had been performed 2 weeks prior at an outside hospital to determine the etiology of cardiomyopathy, and it had shown no significant atherosclerosis. Electrocardiography (ECG) upon admission showed AF with rapid ventricular response and voltage criteria for left ventricular hypertrophy as per Cornell criteria (***Figure 1A***). Although his chest pain resolved, cardiac enzymes revealed acute myocardial injury (troponin I increased from 10 ng/mL to > 40 ng/mL, normal limit < 0.4 ng/mL). Electrocardiography showed globally reduced biventricular systolic function with concentric left ventricular (LV) wall thickness of 17 mm (***Figure 1B***). Cardiovascular magnetic resonance (CMR) imaging confirmed diffusely increased LV wall thickness (maximum thickness 20 mm; ***Figure 2A, B***), severely reduced biventricular systolic function, and diffuse patchy late gadolinium enhancement (LGE) in the left ventricle and both atria consistent with cardiac amyloidosis (CA) (***Figure 2C, E***).

**Figure 3:**
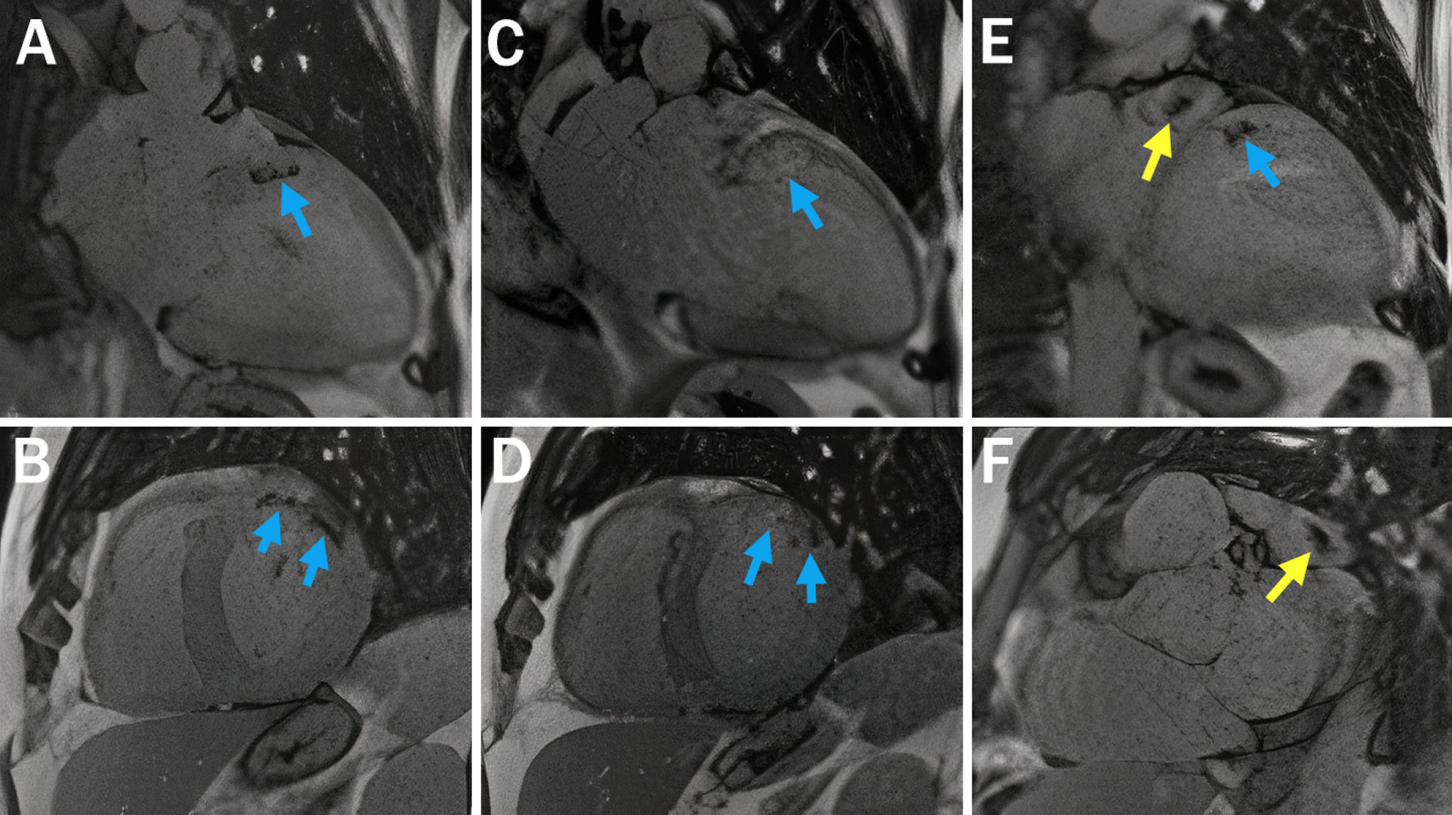
Cardiovascular magnetic resonance imaging with inversion recovery sequence using long inversion time showed area of no contrast uptake at the basal anterior and anterolateral wall (**A, B, E,** blue arrows) that improved on the similar sequence repeated 30 minutes later (**C, D,** arrows) consistent with microvascular obstruction. (**E, F**) Left atrial appendage thrombus (yellow arrow) was also discovered on this sequence. Image enlarged using letsenhance.io.

**Figure 4:**
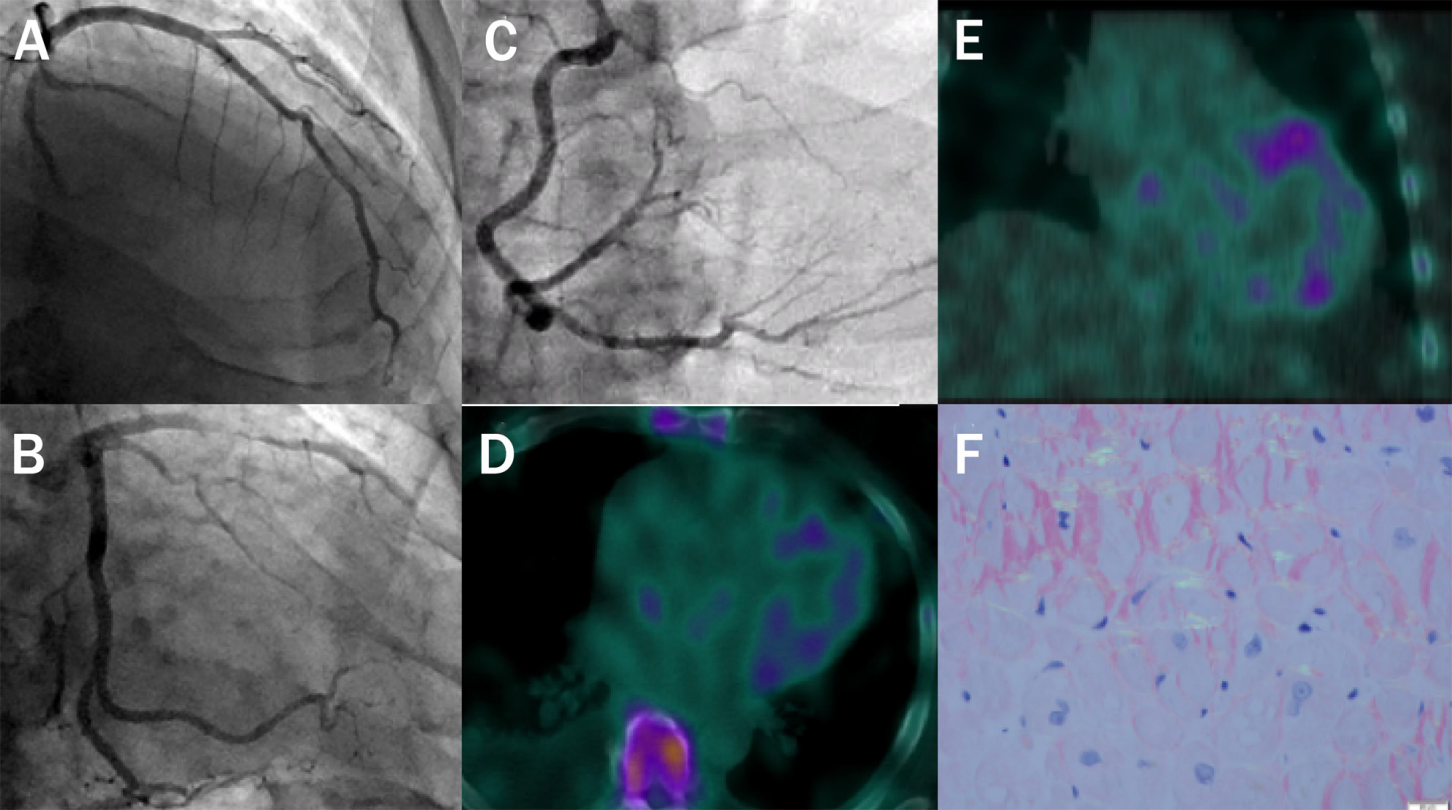
(**A–C**) Coronary angiography showed patent coronary arteries. (**D, E**) ^99m^Technetium pyrophosphate imaging demonstrated significant (grade 2) myocardial uptake. (**F**) Endomyocardial histology with Congo red stain under polarized light showed the characteristic “apple green birefringence” that confirmed diagnosis of cardiac amyloidosis.

Interestingly, CMR also showed dense, focal, transmural LGE in the basal anterior and anterolateral wall (***Figure 2D, E***) with evidence of localized myocardial edema on T2 mapping images (***Figure 2F***) and microvascular obstruction (***Figures 2D, E*** and ***3A–E***), which suggested acute myocardial infarction in the ramus or diagonal branch artery. Additionally, a 1.1-cm thrombus was visualized in the left atrial appendage (LAA) on inversion recovery images using a long inversion time (***Figure 3E–F***). Repeat coronary angiography confirmed patent coronary arteries (***Figure 4A–C***). The cause of the myocardial infarction was hypothesized to be coronary artery embolization from the LAA thrombus. Serum and urine immunofixation excluded monoclonal gammopathy; however, the serum kappa/lambda free light chain ratio was borderline abnormal. ^99m^Technetium pyrophosphate imaging showed significant (grade 2) myocardial uptake (***Figure 4D–E***). Endomyocardial biopsy confirmed transthyretin CA (ATTR-CA; ***Figure 3F***), and genetic testing uncovered a Val122Ile mutation.

The patient was treated with anticoagulation for acute coronary embolism and LAA thrombus. Despite anticoagulation before and throughout hospitalization, he developed an acute ischemic stroke with multiple embolic foci in the right occipital, frontal, and parietal lobes. After the LAA thrombus resolved, the patient underwent successful cardioversion, was started on amiodarone, and improved clinically. He was discharged to a stroke rehabilitation facility with plans to initiate tafamidis (transthyretin stabilizer) and possibly patisiran (transthyretin silencer).

## Discussion

Although hereditary ATTR amyloidosis (hATTR) has been considered rare, its true prevalence is difficult to establish due to under-recognition and variable geographical distribution of causal mutations. Hereditary ATTR transmits in an autosomal-dominant manner but with highly variable penetrance. There are more than 120 identified amyloidogenic mutations, but only a few variants produce the majority of hATTR cases, including Val30Met, Thr60Ala, Ser77Tyr, and Val122Ile (as in our patient).^1^,^2^ An epidemiological study identified the Val122Ile mutation as the most common variant in 3% to 4% of African Americans, with cardiomyopathy as the predominant manifestation.^1^

Our patient had no family history of amyloidosis, had no known extra CA findings, and presented at a relatively young age. In a cohort of patients with increased LV wall thickness, hATTR was found in only 1.6% of patients aged 55 to 64 years but in 11.1% and 11.3% of patients aged 65 to 74 years and 75 to 84 years, respectively.^1^ We did not observe the classic ECG finding of low QRS voltage discordant to LV thickness on imaging in our patient. However, this observation is a relatively late finding in CA, has low sensitivity, and is found in < 40% of patients with biopsy-proven ATTR-CA.^2^,^3^ Our case emphasizes that an absence of low QRS voltage does not exclude CA.

Amyloid infiltration can occur in all cardiac chambers and result in atrial and ventricular dysfunction.^4^ The combination of atrial mechanical dysfunction, enlargement, and fibrillation commonly seen in CA can lead to blood stasis, significantly increasing the risk of thrombosis. Despite being on anticoagulation, our patient experienced an LAA thrombus. In fact, a high prevalence of intracardiac thrombi has been reported in patients with CA. Among 324 consecutive patients with CA who presented for CMR, intracardiac thrombi (90% in the LAA) were found in 13.1% of patients who had AF/flutter even though all were on anticoagulation.^5^ In comparison, the estimated prevalence of LAA thrombi in the general AF population is < 3% when anticoagulated.

System embolization is a known complication of intracardiac thrombus; however, coronary embolism occurs less commonly because the coronary artery anatomy is relatively protected. Coronary embolism is usually under-recognized, accounting for an underlying cause in only 3% of patients with acute coronary syndromes.^6^ Although amyloid infiltration in the intramural coronary arteries can occur, a pattern of regional transmural LGE following a coronary artery distribution favors an embolic etiology after coronary angiography excludes atherosclerosis. Given the challenge of diagnosing coronary embolism, Shibata et al. developed a scoring system to help guide diagnosis.^7^ According to this scoring system, our patient experienced a definite coronary embolus. In a recent systematic review, the left anterior descending artery was the most commonly affected vessel (45.3%) followed by the right coronary artery (15.3%). While not as frequently identified, embolism in the diagonal artery occurred in 5.3% of cases.^8^

To our knowledge, concomitant acute coronary syndrome from coronary embolism and CA has not been previously reported. Additionally, our patient suffered an embolic stroke during hospitalization despite being on systemic anticoagulation. This event underlines the importance of strict anticoagulation in patients with CA and AF regardless of CHA_2_DS_2_-VASc risk stratification.^2^

While advances in noninvasive cardiovascular imaging have improved our ability to detect CA, its diagnosis remains challenging and requires a high index of suspicion.

## Conclusion

Hereditary CA is a relatively rare cause of nonischemic cardiomyopathy. However, patients with CA have an increased risk of intracardiac thrombi even with adequate anticoagulation. Coronary embolism is an unusual cause of acute coronary syndrome but should be considered when coronary arteries are relatively normal and a potential source of embolism is identified.
